# Genome-wide analysis provides insight into the genetic diversity and adaptability of Kazakhstan local goats

**DOI:** 10.1038/s41598-025-02427-8

**Published:** 2025-06-02

**Authors:** Nelly Kichamu, George Wanjala, Kairat Dossybayev, Zoltán Bagi, Bakhytzhan Bekmanov, Szilvia Kusza

**Affiliations:** 1 Centre for Agricultural Genomics and Biotechnology, University of Debrecen, Debrecen, 4032 Hungary; 2 Doctoral School of Animal Science, University of Debrecen, Debrecen, 4032 Hungary; 3 Ministry of Agriculture Livestock, Fisheries and Cooperatives, Directorate of Livestock Development, Sheep and Goats Breeding and conservation Station, P.O. Box 254- 20117, Naivasha, Kenya; 4 Institute of Animal Sciences and Wildlife Management, University of Szeged, Andrássy út 15, Hódmezővásárhely, 6800 Hungary; 5 Livestock, Fisheries, Cooperatives and Irrigation, Directorate of Livestock Production, Ministry of Agriculture, P.O. Box 437-50200, Livestock, Bungoma, Kenya; 6 Institute of Genetics and Physiology, CS MSHE RK, Almaty, 050060 Kazakhstan; 7 Kazakh Research Institute of Livestock and Fodder Production, Almaty, 050035 Kazakhstan; 8 Al-Farabi Kazakh National University, Almaty, 050040 Kazakhstan

**Keywords:** Genetic diversity, Kazakhstan, Local goats, Population structure, Signature of selection, Evolution, Genetics

## Abstract

To understand the genetic structure of local goat populations in Kazakhstan, we present the first comprehensive genomic analysis using a 70 K SNP array, examining 120 samples from six regions. The populations (Kundyzdy, Darbaza, Shokpar, Ushterek, Kenes and Kosseit) are named based on their breeding regions and are referred to ecotypes. Our results present a high genetic diversity, with observed heterozygosity (Ho) ranging from 0.389 to 0.444. Shokpar and Kundyzdy showed excess heterozygosity, while Kosseit and Darbaza had high inbreeding levels. Most populations recorded weak genetic differentiation, except for Ushterek goats, which were more distinct. For adaptability, Runs of Homozygosity (ROH) and Integrated Haplotype Score (iHS) identified 71 genes under selection, with gene ontology enrichment indicating localization as the most represented biological pathway important for cell response. Additionally, over 60% of these genes are co-expressed, suggesting their significant role in survival of these goats in their local environments. Some identified genes, such as *NLRC4*,* HCLS1*, *IL17D*,* IL17RE*, and *IL17RC*, were linked to immune response, an important adaptation trait in the context of climate change. This study not only emphasizes on high genetic variability of local goats in Kazakhstan but also lays the groundwork for developing conservation and breeding programs for local goats in Kazakhstan and other similar agroecological regions for adaptability and resilience under changing environmental conditions.

**Supplementary Information:**

The online version contains supplementary material available at 10.1038/s41598-025-02427-8.

## Introduction

Goats (*Capra hircus*) are said to be descended from the wild bezoar goat (*Capra aegagrus*), which lived within the Fertile Crescent of western Asia (the Iranian region) around 10,000 years ago^1–4^. As humans moved from one place to another in search of fertile places to settle and trade, they brought the goats with them^2^. This movement (migration) led to the emergence of sub-populations and breeds that can be distinguished by different genetic traits and phenotypic expressions^2^. A prolonged stay in new habitats and environments led to adaptations as they experienced various natural selection pressures, this resulted to the development of adaptive genes which enabled them to thrive in these regions. The adaptation process yielded different breeds, sometimes distinguished phenotypically, while other breeds are phenotypically similar but can only be distinguished by molecular data analysis^5^. These variations can be identified by doing signature selection analysis, which highlights genomic regions under natural or artificial selection and provide insights into adaptive evolution and domestication-related traits^6^. For instance, studies on selection signature have identified genes linked to economically important traits, including *HOXA12*,* PPP1CC*, and *LCORL*, which influence morphology, reproduction, and environmental adaptation in some livestock species^7,8^. Currently, the goats have become special livestock species in terms of their importance and adaptation to the environments in which they are produced^9^.

Indigenous goats specifically are preferred due to their unique adaptive features that can withstand various ecological zones^9,10^.They are useful domestic livestock species for rural households to exploit because of their body size, productivity levels, food preferences, and low investment costs^11,12^ and hence they contribute to the livelihood improvement of the rural households by acting as a source of food security and income generation^10,13^ However, the growing human population and the rising demand for livestock products have impacted the genetic diversity of indigenous goats through crossbreeding and the introduction of exotic genotypes^14^.

Kazakhstan, a country that covers the major part of Eurasian Steppe and also situated near the early domestication centers, presents a unique opportunity for studying genetic variability in goats^15^ Besides this, the varied landscape that contains arid, deep valleys and mountainous regions has likely shaped the unique genetic adaptations in local goat populations. This geographical position at the junction of historical migration routes indicates the possible emergence of unique adaptive features in this goat populations^16^. Despite their significance, the local goat populations in Kazakhstan have not been characterized, as they are named after the location or community, they originate from rather than having a specific breed name. Recent population trends in Kazakhstan’s goat sector highlight the urgency of genetic characterization and conservation. Between 2020 and 2024, the national goat population showed fluctuations in numbers, initially decreasing from 2.31 million to 2.32 million head (2020–2022), followed by a recovery phase^17^. These demographic changes highlight the necessity for thorough genetic monitoring and adaptive management techniques to preserve these genetic diversity and population resilience. To worsen the situation, there is little information on genetic diversity and population structure of these indigenous goats, crossbreeding has gone hand in hand with uncontrolled mating. These practices dilute the locally adapted alleles in indigenous populations and over time, such uncontrolled mating can erode unique adaptive features like disease resistance, heat tolerance and compromise the resilience of these populations to climate change and disease outbreaks all these factors limit improvements in genetics, hence further complicating conservation efforts^13^. Most previous studies have focused on mitochondrial DNA and Y-chromosomal markers^15,16^, thus leaving a significant knowledge gap in genome-wide variation patterns. While earlier research successfully utilized microsatellite markers to characterize genetic diversity and population structure in various small ruminant breeds in Eastern Europe^18,19^, recent technological advances have enabled more comprehensive genomic analyses. The use of high-density SNP arrays, such as the 70 K SNP platform, can now provide deeper insights into population structure and genetic diversity patterns which are essential for both conservation efforts and sustainable breeding programs^20^. But it is important to recognize that a lot of population genetic approaches, including those used with SNP data, are predicated on ideas related to the molecular clock hypothesis and the neutral theory of molecular evolution. As more and more empirical data challenges the idea of mutation rate constancy^21^ and shows the ubiquitous role of selection for instance functional mtDNA variation^22^, transcription factor binding by STRs^23^, and non-neutral diversity patterns^24^), these frameworks have come under increasing criticism. New hypotheses that highlight the shortcomings of conventional neutral models include the greatest genetic diversity hypothesis^25^. Therefore, building upon these previous methodological approaches, we used the genome-wide caprine 70 K SNP markers to assess the genetic structure of selected local goat population in Kazakhstan to determine their genetic diversity status using within-population diversity indices including observed and expected heterozygosity (Ho and He), inbreeding coefficients. In addition, the relationship between selected populations was assessed using principal component analysis and phylogenetic trees. Further we identified runs of homozygosity patterns and signatures of selection for local adaptation. While our high-density SNP arrays genomic data fill previous gaps in genome-wide analyses, interpretations of diversity and structure are still problematic, as they may reflect both neutral and non-neutral processes considering changing theoretical paradigms.Results from this study will provides an insight into the genetic architecture of Kazakhstan’s local goat population for improved conservation and management strategies.

## Results

### Genetic diversity indices, ROH and inbreeding coefficients

On average, Ho and He across all populations were similar, with Ho slightly below the expected value. The mean Ho and He for all populations were 0.424 and 0.429, respectively. Population wise, the Ho ranged between 0.389 (Kosseit) and 0.444 (Shokpar), while He ranged between 0.441 (Ushterek) and 0.409 (Kosseit) (Supplementary file1, Figure S1, supplementary material online). All goat populations also registered different figures of inbreeding coefficients, Kundyzdy and Shokpar registered negative FIS indicating excess heterozygosity in both breeds. Inbreeding coefficients also varied across breeds, for instance, Fhom ranged between 0.045 and 0.126, Fhat1 between − 0.002 and 0.135, Fhat2 between 0.002 and 0.084 (Table 1).

**Table 1:** The genetic diversity indices for six local goat populations from Kazakhstan.

Population	*N*	H_o_	He	FIS	Fhom	Fhat1	Fhat2	FGRM
Darbaza	20	0.412 ± 0.16	0.429 ± 0.11	0.052 ± 0.29	0.075 ± 0.09	0.064 ± 0.07	0.076 ± 0.10	0.070 ± 0.08
Kenes	20	0.430 ± 0.15	0.434 ± 0.10	0.004 ± 0.24	0.030 ± 0.03	0.006 ± 0.02	0.034 ± 0.03	0.020 ± 0.02
Kosseit	20	0.389 ± 0.16	0.409 ± 0.12	0.052 ± 0.25	0.126 ± 0.11	0.054 ± 0.08	0.135 ± 0.12	0.094 ± 0.09
Kundyzdy	20	0.426 ± 0.15	0.423 ± 0.11	-0.011 ± 0.23	0.045 ± 0.05	-0.002 ± 0.05	0.053 ± 0.05	0.025 ± 0.05
Shokpar	20	0.444 ± 0.17	0.435 ± 0.11	-0.027 ±0.28	0.006 ± 0.01	-0.007 ± 0.01	0.010 ± 0.02	0.002 ± 0.01
Ushterek	20	0.441 ± 0.15	0.441 ± 0.10	0.000 ± 0.25	0.007 ± 0.04	0.189 ± 0.07	-0.022 ± 0.05	0.084 ± 0.05

Sample size (N), expected heterozygosity (He), observed heterozygosity (Ho), inbreeding coefficient (FIS), inbreeding coefficient based on homozygosity (Fhom), observed homozygosity against whole population (Fhat1), excess homozygosity based on allele frequencies (Fhat2) and identity by descent (FGRM).

**Fig. 1:**
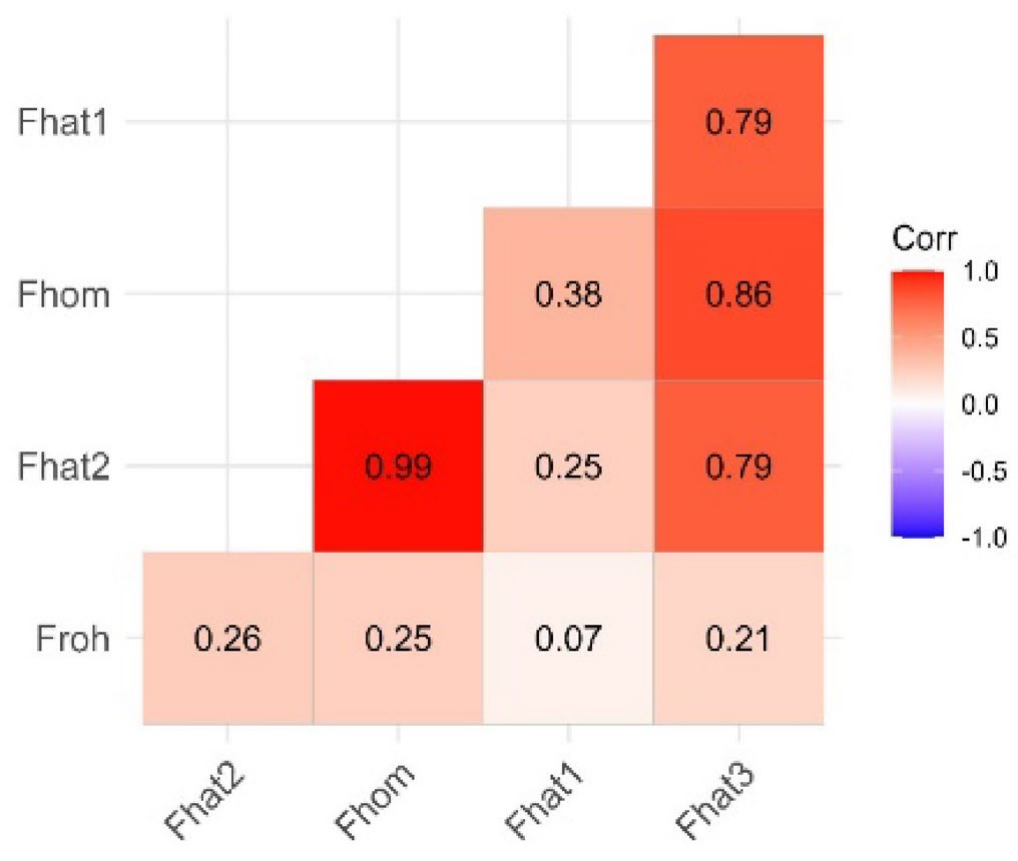
Correlation between inbreeding coefficients for the six Kazakhstan goat populations.

The correlations between different inbreeding coefficients, Fhat1, FGRM or Fhat3, Fhom and FROH are shown in Fig. 1. The highest correlation (0.99) was observed between Fhom and Fhat2 while the lowest correlation of 0.07 between Fhat1 and Froh. Additionally, the correlation between Fhat1 and Fhom as well as Fhat1 and Fhat3 was relatively high 0.86 and 0.79, respectively. We also observed a relatively low correlation between (0.26) Fhat2 and FROH. The high level of correlation between Fhat2 and Fhom indicates that the two methods can provide detailed information on the various aspects of inbreeding as compared to other measures.

Analysis of ROH indicated that the count of short runs (0–6 bps) dominated all others with some population lacking long (> 48) runs, indicating that, most of the inbreeding in these populations happened in recent generations. Kundyzdy and Kenes exhibited highest level count of short runs while Kosseit and Darbaza are the only populations that had longest runs (Table 2).

**Table 2:** Count of runs of homozygosity per breed and length of the run (bps).

Runs	Darbaza	Kenes	Kosseit	Kundyzdy	Shokpar	Ushterek
0–6	1331	2005	1937	2399	1239	1832
6–12	61	19	89	34	2	41
12–24	43	15	78	21	1	3
24–48	19	4	51	9	NA	NA
> 48	7	NA	15	NA	NA	NA

### Intra – and interpopulation relationships and population structure

**Fig. 2:**
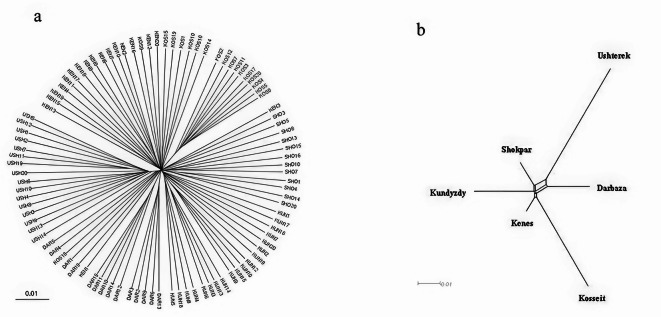
Neighbor joining tree for the studied populations, (**a**) unrooted neighbor joining tree and (**b**) neighbor network based on distance matrix calculated from pairwise Fst values.

To assess both intra- and inter-population relationships, several methods were employed. Intra-population relationships, for example, were examined using a neighbor-joining tree (Fig. 2a). This analysis revealed three main groups, each with several branches or subgroups. These results align with the population grouping shown in Fig. 2b, which divides the populations into five major groups. However, the populations of Ushterek and Kosseit show greater differentiation from the others, while Kenes appears to be less differentiated.

**Fig. 3:**
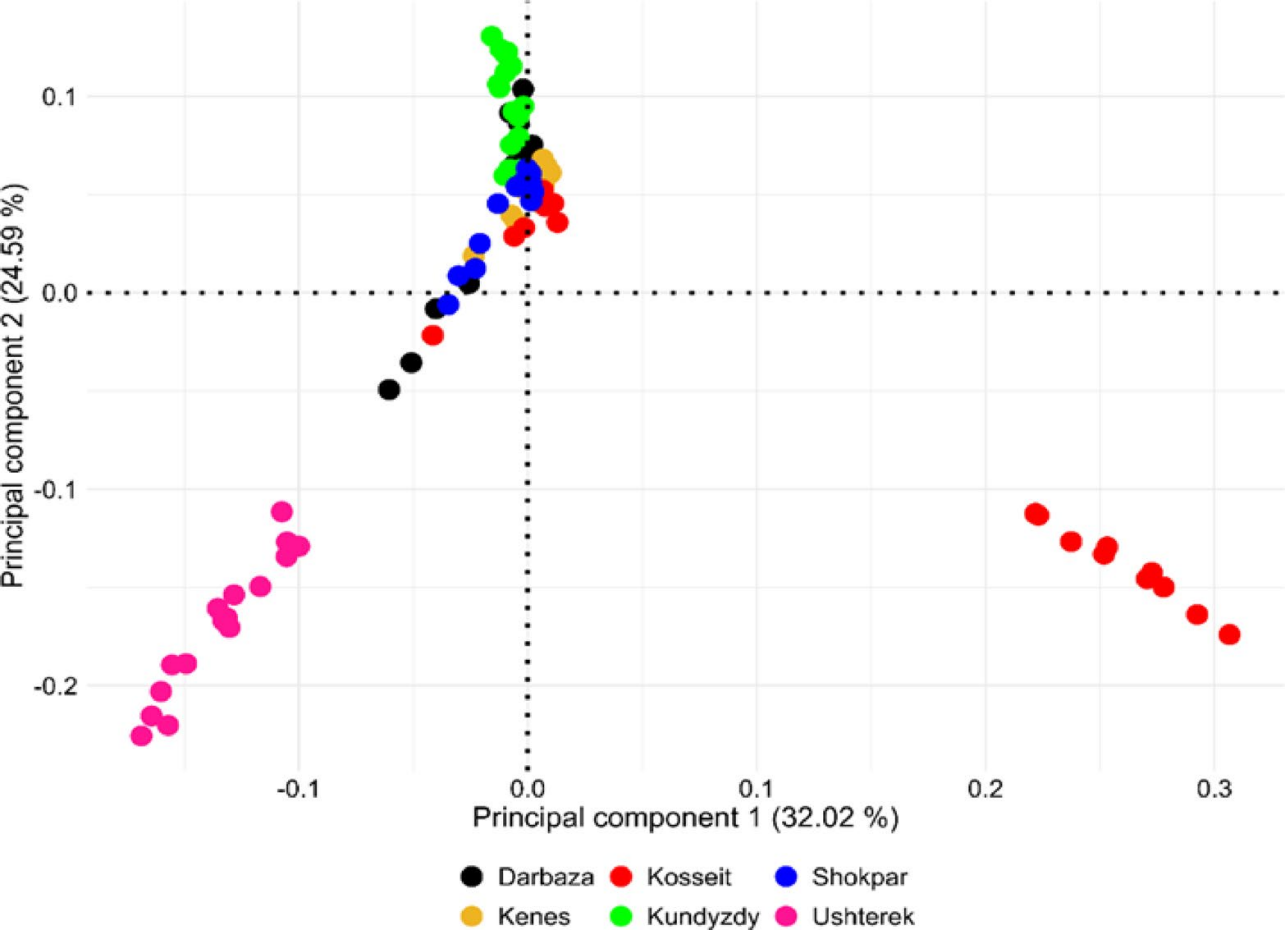
Population structure of six goat breeds from Kazakhstan as defined by principal component analysis (PCA), PC1 versus PC2.

Further, interpopulation relationships were evaluated using PCA (Fig. 3). It was observed that similar to the neighbor-joining trees, three major groups emerged with Ushterek and Kosseit exhibiting a tendency of being genomically distant from other groups, although they appear to be genetically correlated based on PC1. All other populations were clustered together indicating close genetic relationship. Both PC1 and PC2 accounted for more than 50% of the genetic variation between populations indicating the existence of population structure.

**Fig. 4:**
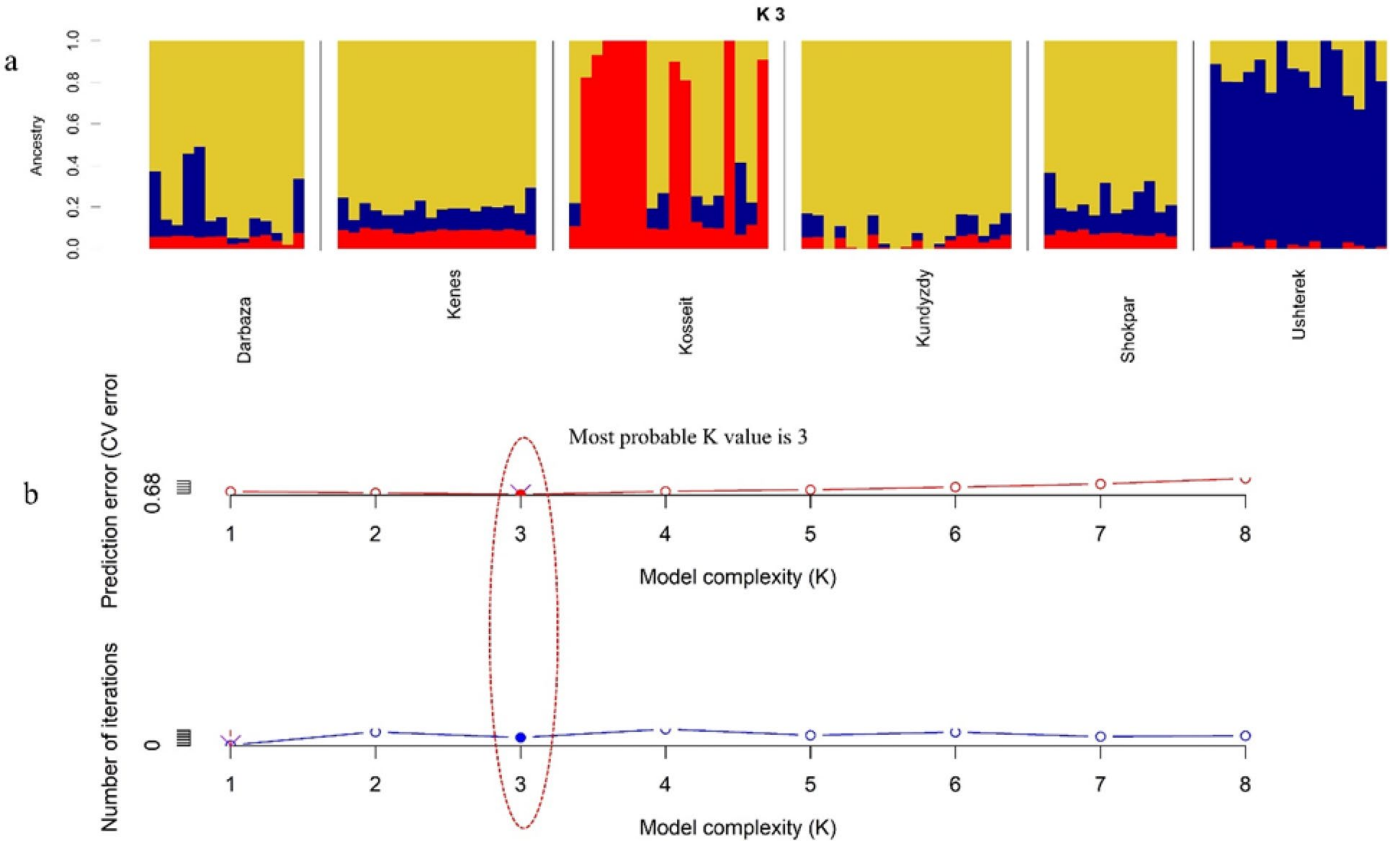
Map of the blood collection points for the local goat population in Kazakhstan generated using QGIS^82^ (**a**) location of the country and (**b**) localization of the sampling area.

**Fig. 5:**
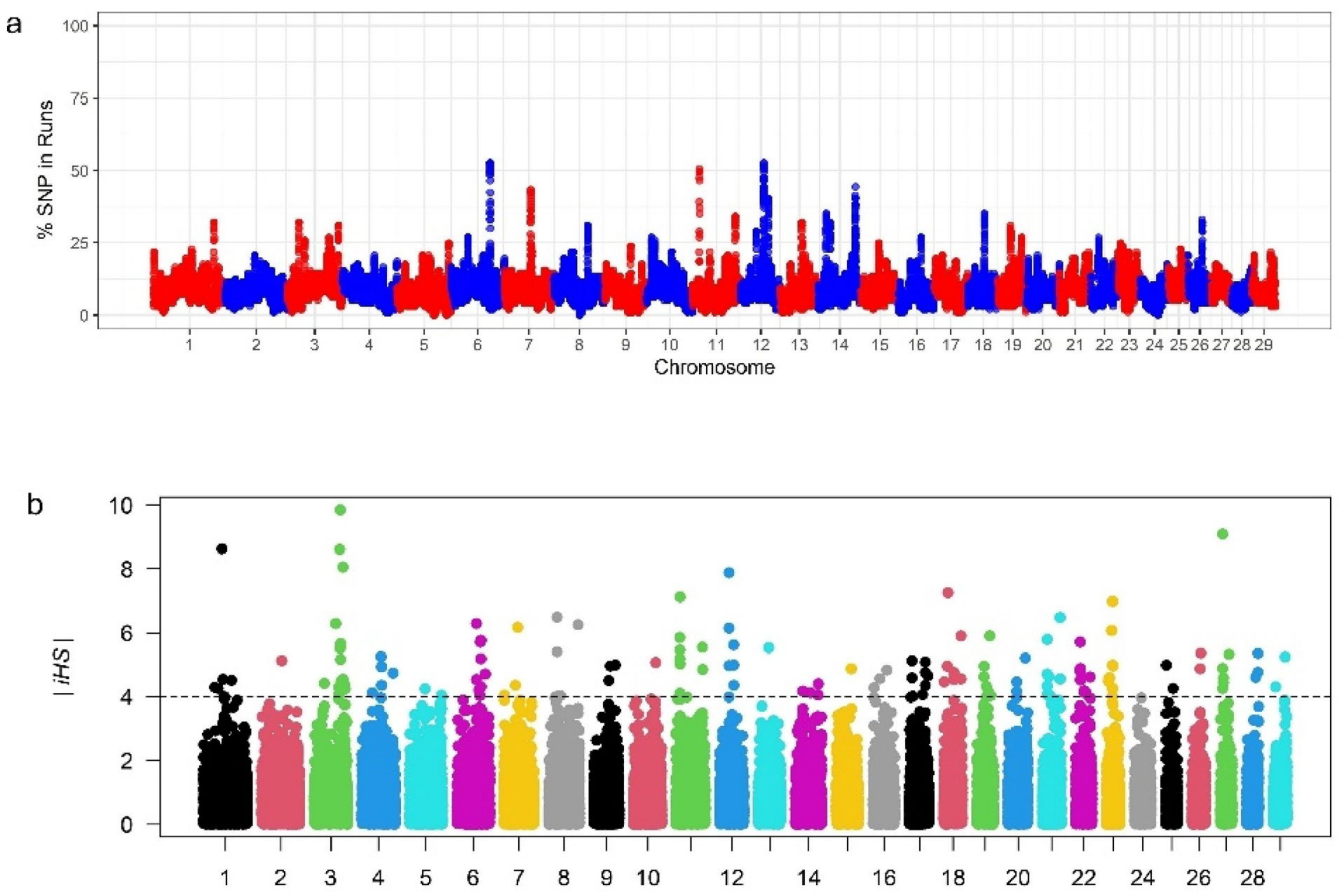
Admixture analysis showing the proportions of ancestral populations for K = 3; (**a**) Bar chart showing the proportion of admixture with each vertical bar representing an individual. (**b**) Cross validation error plot showing the lowest value of CV which corresponds to K = 3.

Co-ancestry analysis was performed using ADMIXTURE software, evaluating various numbers of genetic clusters (K) from K = 2 to K = 8, (see Supplementary File 1, Figure S2, Supplementary material online). The population structure corresponding to the optimal K value is presented in Fig. 4a, while Fig. 4b displays optimal K with the lowest cross-validation error. At this K value, the analysis revealed high levels of admixture, with Darbaza, Kenes, and Kundyzdy showing similar patterns of ancestry, suggesting they represent a single population. In contrast, Kosseit and Ushterek were identified as distinct populations (Fig. 5a, b).

### Identification of signatures of selection

**Fig. 6:**
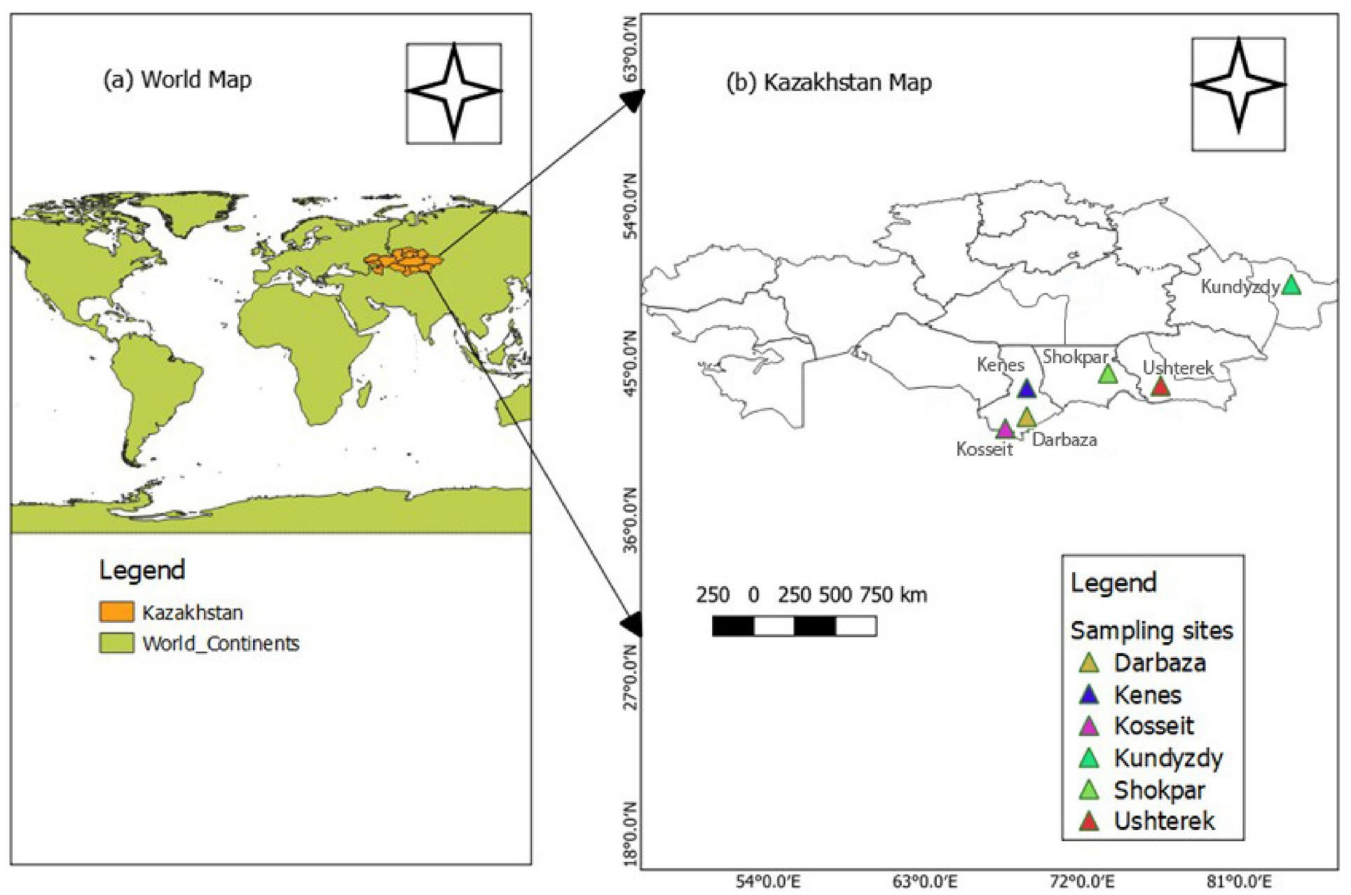
Manhattan plots showing (**a**) percentage of SNPs in runs per chromosome and (**b**) distribution of iHS per chromosome.

Complementary methods were employed to identify selection signatures potentially underlying adaptation to local conditions. ROH identified 98 while iHS identified more than 2000 genes. However, 71 genes were common to both methods, and these were selected for further investigation; (See supplementary File 2, Tables S1 to S3, Supplementary material online). for more details. The distribution of SNPs in ROH and iHS was visualized as shown (Fig. 6a and b).

Gene ontology and enrichment terms and gene interaction network was performed on common genes to have a deeper understanding into the biological functions of identified genes. The most enriched biological function was localization followed by cellular process, developmental process and metabolism which are important functions for species adaptation. On the other hand, gene interaction network revealed that more than 67% of the interaction is co-expression indicating a strong functional relationship between these genes (See supplementary File 1, Figures S3a and 3b, Supplementary material online).

## Discussion

From a total of 120 samples and over 70,000 SNPs, quality control measures yielded a dataset of 97 samples and 49,838 SNPs, achieving an average call rate of 0.97. With a genotyping success rate of 97%, we are confident that our dataset is of high quality, making the conclusions drawn from these results reliable. Genetic diversity studies provide an important insight into the genetic architecture of the population^26^. In our study the mean heterozygosity values (Ho = 0.424; He = 0.429) of Kazakhstan goat populations showed a moderate genetic diversity. Although these measurements of genetic diversity initially seem to be in line with neutral theory, attributes variation to random mutation and genetic rift, new empirical findings cast doubt on this fundamental paradigm. The Neutral theory, for instance, assumes that non-selective processes account for most of the genetic variability. Even in genomic regions that are typically categorized as neutral, there is growing evidence that selection, not drift, significantly shapes genetic diversity in species^22^. For example, genome-wide examinations of humans and water fleas (Daphnia) demonstrate that slow changes in gene versions over generations are caused by natural selection rather than chance. This calls into question the long-held belief that selective pressures do not influence the evolution of DNA sections that do not directly code for proteins^22^.The genetic variation from how findings therefore could be due to the following potential factors: either exposure to intensive selection pressures for economically important traits, or the inheritance of diverse genetic material from their ancestral populations. However, considering that local breeds are rarely subjected to systematic and objective selection programs in traditional management systems, we posit that the observed heterozygosity patterns are more likely to reflect diverse ancestral origins rather than artificial selection effects. This interpretation has historically assumed a neutral baseline, but growing evidence suggests that even SNPs and STRs may overlap with regions under selection^22,23^. For instance, mitochondrial DNA (mtDNA), which was formerly thought to be a neutral marker but is now understood to be functionally significant^22^. The mitochondrial local constraint (MLC) metric further quantifies functional conservation in mtDNA, thereby underscoring how selection shapes even non-recombining genomes^22^The values obtained in this investigation are within those observed in a study on global goat populations. For instance, the study on the worldwide goat population where indigenous goats from Italy recorded (Ho = 0.47; He = 0.397), Mongolian indigenous goats (He = 0.4; Ho = 0.36), Ethiopian Begait goats (He = 0.35; Ho = 0.32), Ugandan indigenous (He = 0.387; Ho = 0.378), and South African indigenous goats (He = 0.310 ; Ho = 0.330)^27–30^.It is opined that these heterozygosity ranges fall within the sustainable genetic breed management, and thus we conclude that the studied populations have a considerable within-population genetic diversity.

The current FST values of the population (0.014–0.052) show moderate genetic differentiation. This are similar to the findings of the VarGoats project, which reported generally low FST values (0.017) in Asian goat populations^31^. 0.024 for Bulgarian native goats^32^ 0.016 in Ugandan Indigenous Goats^33^, These values suggest that even though there is some genetic differentiation, there still exists some levels of gene flow among these populations that may have a raised due to pastoralist mobility nature. However, the assumption that low FST reflects neutrality is increasingly questioned. Adaptive introgression, or balanced selection, can preserve functional diversity across populations while reducing differentiation, as recent research shows that gene flow and selection are not mutually exclusive^21^. The analysis of FIS also revealed a concerning pattern in the Kosseit population (-0.011 to 0.052). While some subpopulations showed a healthy genetic mixing through negative FIS values, the Kosseit group showed a clear sign of isolation with reduced genetic variability (Ho = 0.389 ± 0.16), high levels of inbreeding based on homozygosity values (Fhom = 0.126 ± 0.11) and positive inbreeding coefficient (FIS = 0.052). Although drift and isolation are reasonable explanations, it is impossible to deny the influence of selection on inbreeding patterns. For instance, inbreeding may purge harmful recessive alleles, a process that cannot be detected under rigorous neutral models^21^ .From our study, the isolation appears to result from multiple factors including geographical barriers which tends to limit the gene flow into the population, reduction in effective population size due to selective breeding practices, and the use of related breeding bucks within the community. Similar patterns of inbreeding have been documented in other goat populations for instance, Ugandan indigenous goats (FIS = 0.014), Chinese indigenous goat populations (FIS = 0.03) and Sudanese Nubian goat breed (FIS = 0.073)^33–35^, study on four cattle breeds using 15 STR loci also reported an (FIS = 0.013)^38^, where small population size, traditional management practices, cultural preference to specific trait or colours, intensive selection and geographical barriers were identified as reasons for the levels of inbreeding observed. For our study, the impact is particularly concerning given the challenging environmental terrains where these goats have to thrive for survival.

ROH islands identified in certain genomic regions indicate that some specific alleles have been fixed in the populations, presumably due to selection or genetic drift^26^, thus suggesting adaptation to local conditions. The length and distribution of ROHs offers an important insight into population structure and selection processes. Long ROHs indicate the presence of a more recent inbreeding, while shorter ROHs suggest the presence of ancient inbreeding within a given population^36,37^. The mean sum of ROH segment coverage was generally higher for short ROHs than for long ROHs. However, Kundyzdy populations showed a higher average sum of short run at ROH > 6mb^38,39^,. The distribution of ROH coverage reported in this study agrees with findings from other studies in goats. For instance^31^, on their study in various goats in Switzerland observed more of short run ROH segments (< 1mb) than those that were (> 6mb), Egyptian goats breed more of < 2mb^40^, Italian goat breeds that were raised in the isolated northern Italy^41^, and Yunan goat breeds in China^42^, indication that the studied goat breed had more ancient inbreeding event probably due to a more distance common ancestor.

This highest count of short ROH in the Kazakhstan studied goat populations indicates that they could have been initially established by small founding populations but were not particularly highly affected by recent inbreeding. Selection also plays a role in the frequency of ROH in the genome. This finding aligns with the notion that certain populations can maintain genetic diversity despite geographic isolation and that ROHs do not only arise because of inbreeding, but also can provide information about demographic events and selective sweep^43^.

The use of Neighbor-net joining (Fig. 2b) to assess interpopulation relationship revealed a complex pattern of genetic relationship among the studied goat populations. There was a close relationship between five populations except for Ushterek goats which had a distinct cluster, indicating a unique evolutionary history. This observation is also enhanced in the admixture analysis results (Fig. 5) where high levels of admixture between the studied populations were observed, with Darbaza, Kenes and Kundyzdy showing similar patterns of admixture. This suggests the presence of a single population which may have occurred due to historical crossbreeding amongst them. The country’s traditional transhumance routes, which have historically made it easier for animals to travel between different regions, may have contributed to this degree of genetic admixture between populations^44^. Additionally, the sale of breeding bucks, the cultural practices of different ethnic groups in the region^45^, especially their traditional nomadic way of livestock breeding and husbandry techniques and the modern breeding initiatives that have brought about novel genetic exchange patterns, though these are often more structured and documented than historical movements. Together, these factors create a complex web of genetic relationship which has shaped the current population structure in the region.

The historical background of genetic connectivity between populations can be traced through multiple lines of evidence. The findings are similar to those observed in other regions, like the admixture recorded in Ugandan indigenous goats^33^. Djallonke and Sahelian goat breeds in Benin^46^, and in new world goat populations where Asian, African and European goats showed evidence of admixture^47^ The VarGoats project has further illuminated these historical admixtures by demonstrating how Y-chromosomal haplogroups reflect ancient migration patterns and more recent breeding practices^31^. The Kosseit population showed a high level of single ancestry component, indicating significant geographic isolation, which is supported by high Fhat2 and FGRM values. These findings are in line with other goat population studies such as Bohuai goats in China^48^ and Mongolian cashmere goats^49^. Where their genetic structure was shaped by geographical isolation, breeding and husbandry practices. Various factors may have contributed to results observed in Kosseit goat population, this involves the Kazakhstan geographical terrain (mountains and deep valleys) for example the presence of the Tien Shan and Altai mountains may have created barrier that restricted the free movement of animals in the region, lack of access to breeding centers might have also caused a challenge in the introduction of new breeding material in the population^50^. Besides, the Traditional livestock production systems employed which are passed down through generations might have encouraged inbreeding within the same populations. Additionally, selection for a trait of importance within the locality has further focused on a specific genetic characteristic^51^. The findings are in line with other goat studies such as Ethiopian Begait goat^28^, and Bulgaria native goats^32^, which observed the mentioned factors as the influence on their single co-ancestry structure.

For this, the sustainability and adaptability of any population can only be maintained by understanding its genetic diversity^52,53^. The different patterns between populations with lower genetic diversity (Kosseit and Darbaza) and those with higher genetic diversity (Shokpar and Ushterek) underscore the significance of balanced conservation strategies. Some populations have low genetic differentiation because of long-term selective pressures and management systems. This is brought about by the limited gene flow and traditional breeding methods used. Understanding these complex relationships and their historical context is essential to developing effective conservation strategies. As it is important for long-term survival and adaptability to ever changing climatic conditions. Therefore, it is important to prioritize those populations that showed some signs of genetic isolation or low genetic diversity by maintaining its genetic variability.

In developing countries such as Kazakhstan, productivity and reproduction in livestock population is generally affected by the environmental conditions, high temperatures, rocky and mountainous topography, disease incidences, low- and poor-quality feeds and pastures and inadequate information in livestock management^54^. To withstand these challenges, species must develop some adaptive mechanisms that should enhance their immune response, growth, and overall livestock welfare^55–57^. The existence of mountainous terrain and varied ecological zones in Kazakhstan may have helped in shaping the genetic diversity and enhancing the adaptability mechanism of their local goat populations^16^. Besides this, the physical barriers created by high-altitude ranges and deep valleys have led to the restriction of livestock movement thus affecting the genetic diversity of goat population in the region^15^, making geographical isolation become a possible key factor to the genetic differentiation observed among the studied goat populations. Additionally, traditional pastoral farming, including seasonal migration along interconnected routes, have further facilitated the gene flow and the exchange of breeding males, thus influencing the genetic landscape of the goat population^45^.

To withstand these challenging conditions, these goats may have developed special mechanisms to boost their adaptation. In our study, we identified various candidate genes that were suspected to be underpinning adaptation to local environmental conditions. Here we only discuss genes that were detected by both iHS and ROH methods. The results from the gene ontology enrichment analysis revealed that the most over-represented biological term was localization (see Supplementary File 1, Figure S3a, Supplementary material online). Besides, gene-gene network analysis showed that 67% of these genes interact by co-expression (see Supplementary File 1, Figure S3b, Supplementary material online, ). This suggests that these goat populations may have undergone evolutionary adaptations, leading to the development of proteins that localize to various cellular compartments, potentially evolving new functions that enhance their survival in harsh environmental conditions. In general, the most over-represented biological pathways identified in our study, including localization, cellular processes, and developmental processes, have also been reported in other research works. Notably, these pathways were observed in studies by^58^ in Ethiopian goat populations, and^18^ in Swiss Alpine goat breeds. This consistency confirms the generality of these adaptation patterns across different ecological zones.

Some of the genes that we identified and are suspected to be enhancing these goats’ adaptability include *BIRC6*,* IFT88*,* IL17D*,* IL17RE*,* IL17RC*,* NRN1L*,* NLRC4*,* HCLS1*,* TSHR*,* FBXO40* and *GJB2* (See supplementary File 2, Tables S1 to S3, Supplementary material online), which have been known to play a key role in immune response, metabolic regulation, skeletal and muscle development and reproductive efficiency. For instance, *NLRC4* and *HCLS1* genes play a key role in immunity function by acting as a sensor which recognizes some bacteria components and activates inflammasome pathways to produce pro-inflammatory cytokine which is an essential response for goat populations that are produced in regions that are susceptible to bacterial infections and diseases infestation^59^. Additionally, *HCLS1* has been associated with resistance to *E.coli* infections in sheep^59^ and thus highlighting its importance in livestock immunity. Further, we also identify *IL17D*,* IL17RE* and *IL17RC*, gene which is part of interleukin-17 group, this gene act by regulating the cytokine production and influence the recruitment of innate immune cells to infection areas^60^.For instance In humans, the IL17RE gene have been seen to work in conjunction with IL17RA to drive IL-17 C-mediated mucosal and TH17 immune responses^61^, These interactions are important as they play a role of enhanced immunity^62^. Similar mechanisms have also been observed in identified in teleost fish like turbot^63^. Besides, studies have also reported the importance of this gene in regulating immune system in including the Ethiopian indigenous goats populations^64^, East African goats in Uganda^32^ and Garfagnina goats breeds in Italy^65^ and have also been associated with gastrointestinal parasite resistance in sheep^59^. Maintaining the health of the animals is one of the important economic aspects in livestock production, especially those produced in regions prone to disease pathogens^54^.Hence the presence of these immune-related genes suggests that enhanced immunity is an important trait under selection in Kazakhstan goat populations.

*BIRC6* and *TSHR* genes are said to be responsible for maintaining pregnancy health in bovine^66^. Some studies have linked *BIRC6* to the increased litter sizes, hair follicle development and reproductive efficiency for example in the Mongolian cashmere and Markhoz goats^67,68^. The role that this gene plays in the reproductive trait makes it a valuable genetic marker to be involved in the breeding program especially for the regions whose fertility is the key factor to livestock breeding. Besides, the *TSHR* gene identified in our study is responsible for signal transduction, specifically in seasonal reproduction and cell survival which are vital in adapting to reproductive strategies in various environmental signals^69^. The genes have been observed to influence seasonal reproduction in livestock, for instance, the presence of *TSHR* was seen to have influence on reproductive seasonality in Swiss goats^70^. Additionally, this gene has been linked to metabolic regulation and photoperiodic control of reproduction, enabling all year-round egg production in chicken^71^. In sheep, TSHR has been associated with environmental adaptability and litter size^72–74^.

The *IFT88* gene is essential for the formation of cilia and flagella, making it important for various cellular processes, including signaling and movement. As a result, it plays a significant role in development and cellular homeostasis^75^. In goats, the *IFT88* gene has been associated with cashmere and fiber production, hence we propose that this gene may have undergone unconscious selection pressure. This productivity is a key economic trait valued by many livestock keepers in relevant regions, making it an important focus in breeding programs. Breeders aiming to enhance fiber and cashmere yields should prioritize this trait in their selection strategies^67,76^. Another gene identified in our analysis was *FBXO40* gene which is vital in cell survival and proliferation acts by controlling the pathways related to cell growth and cell death mechanisms^77^. In livestock, the *FBXO40* gene has been related to muscle formation and control^78^. The *FBXO40* gene has been observed to enhance muscle development in pigs^79^. For Kazakhstan goats, we propose that this gene is crucial for physical endurance thus important in moving across the steep valleys and mountainous regions of Kazakhstan. On the other hand, the genes *GJB2* and *GJB6*, which encode gap junction proteins associated with cell communication, were also identified. These genes influence body size, skeletal and embryonic development, as well as indirectly affecting growth traits^80^.

### Management implications and future conservation perspectives

Our results have important implications for conservation and breeding programs and indicate the need for further studies. The Kosseit population emerges as priority for conservation efforts owing to their low levels of genetic diversity and high inbreeding coefficients. However, maximum genetic diversity theory (MGD) cautions against simplistic interventions thus maintaining genetic diversity is not merely about avoiding inbreeding but also conserving the adaptive traits through selection driven variation^24^. Therefore, Important interventions like adoption of a controlled breeding program and periodic introduction of unrelated breeding bucks should be considered to cub further genetic erosion in this population. On the other hand, the high genetic diversity exhibited in the Shokpar and Ushterek goat populations act as a major genetic reservoir that needs to be carefully monitored and maintained for their genetic variation. Genetic polymorphism studies in Eastern European breeds have revealed important insights into breed characterization and genetic diversity patterns^81^, highlighting the value of molecular markers for understanding breed relationships and developing conservation strategies. The selection signatures identified in this study will serve as important features in target breeding programs that will seek to improve adaptation while maintaining genetic diversity. However, there are several limitations in our current study that need to be addressed through future research. The relatively small sample size per population might be responsible for the inability to discern some rare variants and fine-scaled population structure. Also, the absence of phenotypic data at this time hinders any attempts to make such relations between the genetic variants detected and adaptive traits. A deeper understanding could be gained by incorporating some details as the correlations of environmental data and genetic patterns which can explain the local adaptation mechanisms in detail. To rule out any ambiguities between neutral and selective processes, future studies should consider incorporating temporal genomic data such as tracking allele frequencies across generations and environmental covariates. Whole genome sequencing can be done to identify functional variants overlapping within the neutral SNPs while including a detailed phenotypic characterization, and environmental data. Such an inclusive approach will allow for a better understanding of the potential adaptive traits of these goat populations and inform on better strategies on how conservation measures can be put in place. The absence of phenotypic data at this time hinders any attempts to correlate genetic variants with adaptive traits. Additionally, monitoring and evaluation measures should be put in place to help in assessing the changes in genetic diversity and population structures especially for those goat populations that have been identified as being at risk of extinction. This will facilitate utilization in decision making about the verification of breeding outcomes by contributing value to the generation and preservation of genetic and phenotypic information in a central database. Furthermore, successful implementation of conservation strategies requires a strong stakeholder engagement and coordination programs. Which includes establishing networks between breeding centers, research institutions, and local farmers organizations, developing training programs for genetic management, and creating a regional conservation action plan with clear timelines and responsibilities. Regular monitoring and evaluation of conservation outcomes will be essential to assess the effectiveness of these interventions and make necessary adjustments. These evidence-based conservation strategies, derived from our genomic findings, provide a framework for maintaining the genetic integrity of Kazakhstan’s local goat populations while preserving their adaptive potential for future breeding programs.

## Conclusion

Based on the high-density 70 K SNP data, this study therefore presents a comprehensive genomic characterization of the local goats of Kazakhstan, which not only unveils important patterns of genetic diversity, population differentiation but also potential adaptive traits. While this advanced methodology provides high-resolution insights, our interpretations remain constrained by reliance on neutrality-based metrics such as FST and ROH, that is rooted in the flawed assumptions of neutral theory and the molecular clock hypothesis. The results of the molecular assessment revealed a high variability in levels of genetic diversity among populations, with Kosseit and Darbaza exhibiting alarming levels of low genetic diversity and high-risk rates of inbreeding which increases susceptibility to disease and low reproductive performance, indicating an urgent need for conservation efforts targeting these populations. On the other hand, the genetic diversity of the Shokpar and Ushterek populations is higher, making them reservoirs of rare alleles for the breeding program aimed at enhancing resilience while preserving genetic variation. The identification of selection signatures and adaptive genes (NLRC4, TSHR, IFT88) linked to immunity, reproduction, and environmental stress underscores the pervasive role of selection in shaping genomic architecture of Kazakhstan’s indigenous goats, although our finding is incompatible with the neutral theory of drift-dominated evolution, but it provides an overview of population structure of this population. Gene Ontology (GO) enrichment highlighted important pathways including localization and cellular processes, suggesting that the protein localization mechanism is critical for survival in harsh environmental conditions which aligns with growing evidence that functional constraints, even in non-coding regions, challenge neutrality-based frameworks. While the use of high-density 70 K SNPs in our study provides high-resolution insights, our interpretations remain constrained by reliance on neutrality-based measurements which are rooted in the flawed assumptions of neutral theory and the molecular clock hypothesis, which recent studies has mostly rejected due to their predictive nature. Therefore, the use of Maximum Genetic Diversity (MGD) theory, which posits that diversity is actively maintained to buffer environmental change, has the potential to provide a more robust lens for interpreting genomic patterns in various species. Basing on this argument, this study therefore contributes significantly to understanding the indigenous goats genetic resource in Central Asia, yet its broader implications focused on adopting selection-aware models. Future work should therefore consider integrating whole-genome sequencing, phenotypic data, and Maximum Genetic Diversity (MGD) theory to disentangle adaptive processes from outdated neutral assumptions. By bridging these methodological advancements with theoretical innovation, we can develop strategies that will conserve both genetic diversity and adaptive potential of this indigenous populations in various agroecological zones worldwide.

## Materials and methods

### Sample collection and genotyping

A total of 120 blood samples were collected from different villages which located in South (Darbaza, Shokpar, Ushterek, Kenes and Kosseit) and East (Kundyzdy) parts of Kazakhstan. To study 20 individuals were randomly selected from each village (Fig. 4).

**Fig. 7:**
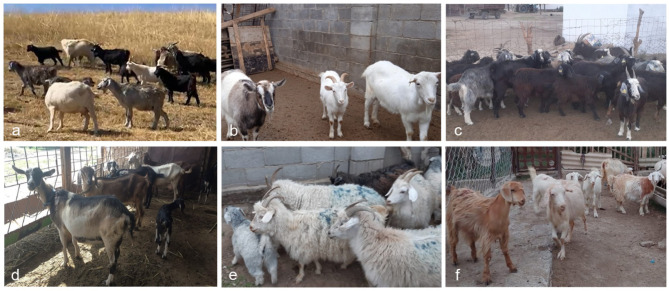
Local goats sampled in Kazakhstan; (**a**) Darbaza, (**b**) Ushterek, (**c**) Kosseit, (**d**) Shokpar, (**e**) Kundyzdy, and (**f**) Kenes (Photos taken by authors).

The studied goat populations are not formally characterized as distinct breeds but rather represent ecotypes of local aboriginal breeds, here named after the villages where they are raised. These goats exhibit several phenotypic manifestations such as ear set, horn shape, and coat colors, reflecting the lack of systematic breed classification in Kazakhstan and demonstrates phenotypic diversity (Fig. 7).

Samples were collected by experienced veterinarians, following all requirements. Blood samples were collected from the jugular vein of the goats by using vacutainer tubes^83^. The samples were then stored in the laboratory freezer at temperatures below − 20 °C, awaiting to be processed to FTA cards^84^ and sent to the NEOGEN Company in Scotland for genotyping using CaprineSNP70K Bead Chip array (Illumina, USA). The 70 K bead chip contains more than 70,000 evenly spaced SNP-targeting probes, which allows adequate SNP density for assessment of genetic diversity and signatures of selection. The whole pipeline from DNA isolation to variant call was performed by the genotyping company. Post-genotyping data management was performed interchangeably using PLINK v1.9 and v2^85^ The data used in this analysis before quality control comprised 120 samples, each with 64,264 autosomes. Quality control was done using the following filtering thresholds:

- Exclusion of samples with more than 10% of missing data,
- SNPs with minor allele frequencies of less than 5%,
- SNPs with more than 10% missing genotypes,
- SNPs which violated Hardy-Weinberg equilibrium (HWE) using “--hwe 1e-6 keep-fewhet” flag.
- We excluded first-line relations (parent- offspring and sibling-sibling) using --king-cutoff 0.177.
- All SNPs at linkage disequilibrium were pruned using the “--indep-pairwise 200 kb 1 0.5” flag and options.

During the quality control process, the following exclusions were made:

- One sample was removed due to missing genotypes,
- 2,059 variants were excluded because of missing genotype data,
- 8,588 variants were discarded for not meeting minor allele frequency thresholds.
- 22 samples were removed due to close relationships,
- 3,711 variants were eliminated based on LD pruning thresholds,
- 58 variants were excluded due to HWE threshold violations.

Quality control measures resulted in a dataset comprising 97 samples and 49,838 SNPs, with an average call rate of 0.97 for further analysis.

### Ethics declarations

Sampling in this study were approved by Local Ethics Commission Institute of Genetics and Physiology CS MSHE RK, approval number #3, 19.09.2024. All methods were carried out in accordance with relevant guidelines and regulations established by the institutional committee and in compliance with national legislation on animal experimentation. Furthermore, all methods are reported in accordance with ARRIVE guidelines to ensure comprehensive and transparent reporting of animal research. The welfare of the animals was monitored with appropriate measures implemented to minimize discomfort and distress.

## Bioinformatic analysis

### Within and between population genetic diversity

Basic diversity indices, including observed and expected homozygosity (H_e_ and H_o_) were calculated using Arlequin software^86^, a standard tool for estimating genetic diversity indices. while the inbreeding coefficient (method-of-moments F coefficient estimates calculated as (observed homozygote count - expected count) / (total observations - expected count) was calculated by Plink v 1.9,this allows for a straightforward estimation of excess homozygosity in a given population. It is important to mention here that this F is later referred to as Fhom. To identify the genetic structure and relationships between populations, several methods were applied. Firstly, multidimensional scaling was performed by principal component analysis (PCA) using Plink v2,PCA was chosen as it is appropriate for reducing dimensionality and visualizing genetic variation, under the assumption of linear independence between principal components. Visualization of the PCA was done using tidy verse package^87^ in R software^88^. Secondly, pairwise fixation index was performed using StAMPP package^89^ implemented in R^88^, this allows for biallelic SNP data and allows for estimation of population differentiation under the assumption of random mating within populations, and it was performed to determine the level of differentiation between ecotypes. Neighbor joining network was constructed using Split Tree program^90^, These methods rely on genetic distance matrices derived from SNP data and do not assume Hardy-Weinberg equilibrium, making them suitable for diverse population structures. Further, the relationship between individuals within the population was calculated and visualized using Tassel software^91^. Admixture proportion or ancestry analysis of each sampled individual was performed using algorithm implemented in ADMIXTURE 1.3 software^92^, This method assumes Hardy-Weinberg equilibrium and linkage equilibrium within clusters. The algorithm was performed in unsupervised clustering with number of clusters (K) set between 1 and 8. The optimal K value was identified as the K value with the lowest cross-validation error. Visualization of Admixture results was performed using BITE v2^93^ package implemented in R software^88^.

### Detection of runs of homozygosity (ROH) and inbreeding

Data used to detect ROHs was not trimmed for linkage disequilibrium (LD) since LD is part of the genomic variability that defines phenotypic variations between individuals and populations. ROH was done in R package – detectRUNS^94^ using sliding window method by applying the following thresholds: window size of sliding window of 15, the threshold of overlapping windows of the same state (homozygous/heterozygous) to call a SNP in a RUN-0.05, minimum number of SNPs in the run-3, minimum number of homozygous/heterozygous SNP in the sliding window-1, minimum length of run in base pairs (bps) -1000 and minimum density of SNPs per 1000 bps = 1000. The detected runs were grouped into five depending on the length; 1–6 bps, 6–12, 12–24, 24–48 and > 48bps.

### Identification of signatures of selection

The identification of ROH islands was performed using the detectRUNS R package^88^. ROH islands (top runs) were defined as region where SNPs are inside runs in more than 70% of the individuals in the population. And it is believed that genes identified within these ROH islands underpins adaptation to local environments. Further, a complimentary method to ROH islands - Integrated haplotype score (IHS) method was used to detect signatures of selection using Rehh package^95^ implemented in R software. Candidate regions were calculated and identified using a sliding window method by applying thresholds similar to ones applied in the identification of ROH islands. BioMart package, developed under Bioconductor, and implemented in R software was used to hunt for protein coding genes at or near the genomic regions identified as signatures of selection. Gene annotation was done based on ARS1.2 annotated genome^96^ on both + and – strands. Only genes that were found in both ROH Islands iHS method were considered for further gene ontology terms and network analysis. Gene ontology terms were performed using Metaphase platform^97^, while gene-gene network analysis was performed by GeneMania^98^. Description of gene functions was done using the already published research works.

## Electronic supplementary material

Below is the link to the electronic supplementary material.

Supplementary Material 1

Supplementary Material 2

## Data Availability

Data supporting this manuscript is available upon request from the corresponding author.
