# Supplementary material for: Genome-wide analysis provides insight into the genetic diversity and adaptability of Kazakhstan local goats
Source: Sci Rep. 2025 Jun 2;15:19327. doi: 10.1038/s41598-025-02427-8 (PMC12130542; doi:10.1038/s41598-025-02427-8)
Supplement: Supplementary file 1 — Supplementary Material 1 [file 41598_2025_2427_MOESM1_ESM.docx]

**Supplementary File 1**

**Genome-wide analysis provides insight into the genetic diversity and adaptability of Kazakhstan local goats**

Nelly Kichamu^1,2,3^, George Wanjala^4,5^, Kairat Dossybayev^6,7,8^, Zoltán Bagi^1^, Bakhytzhan Bekmanov^6,8^, Szilvia Kusza^1,^*


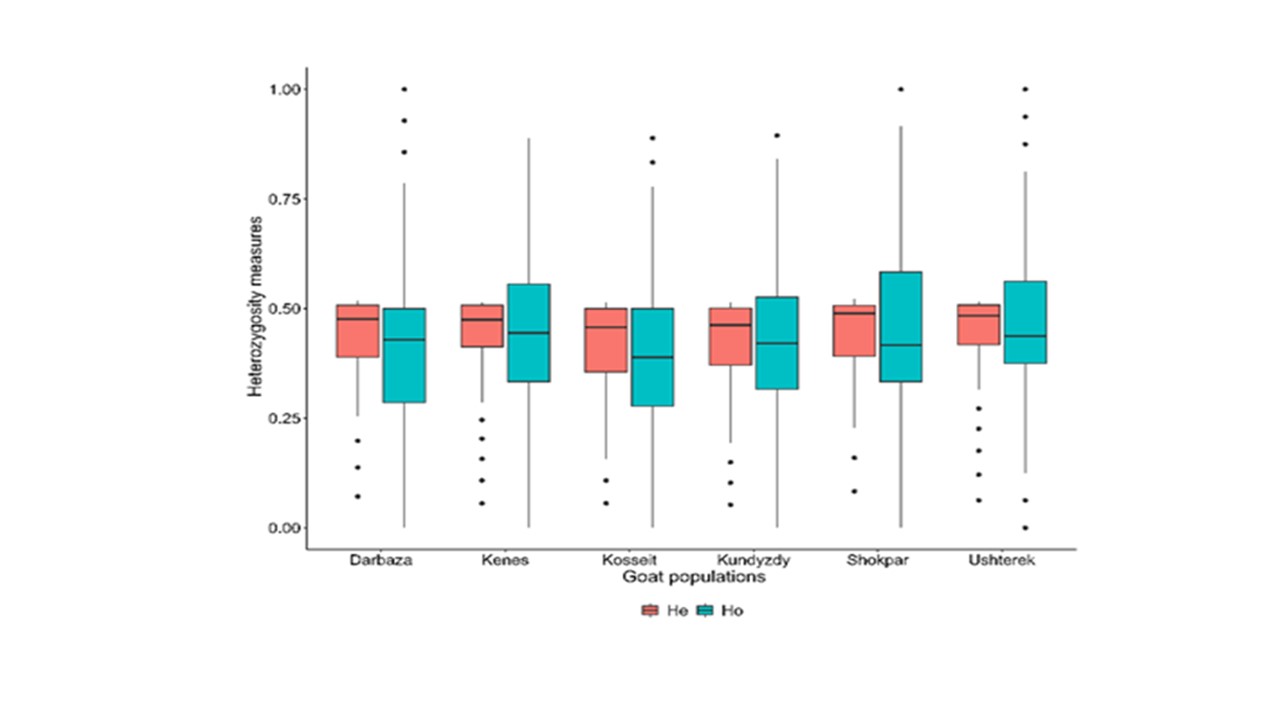


**Figure S1:** Observed (Ho) and expected (He) heterozygosity


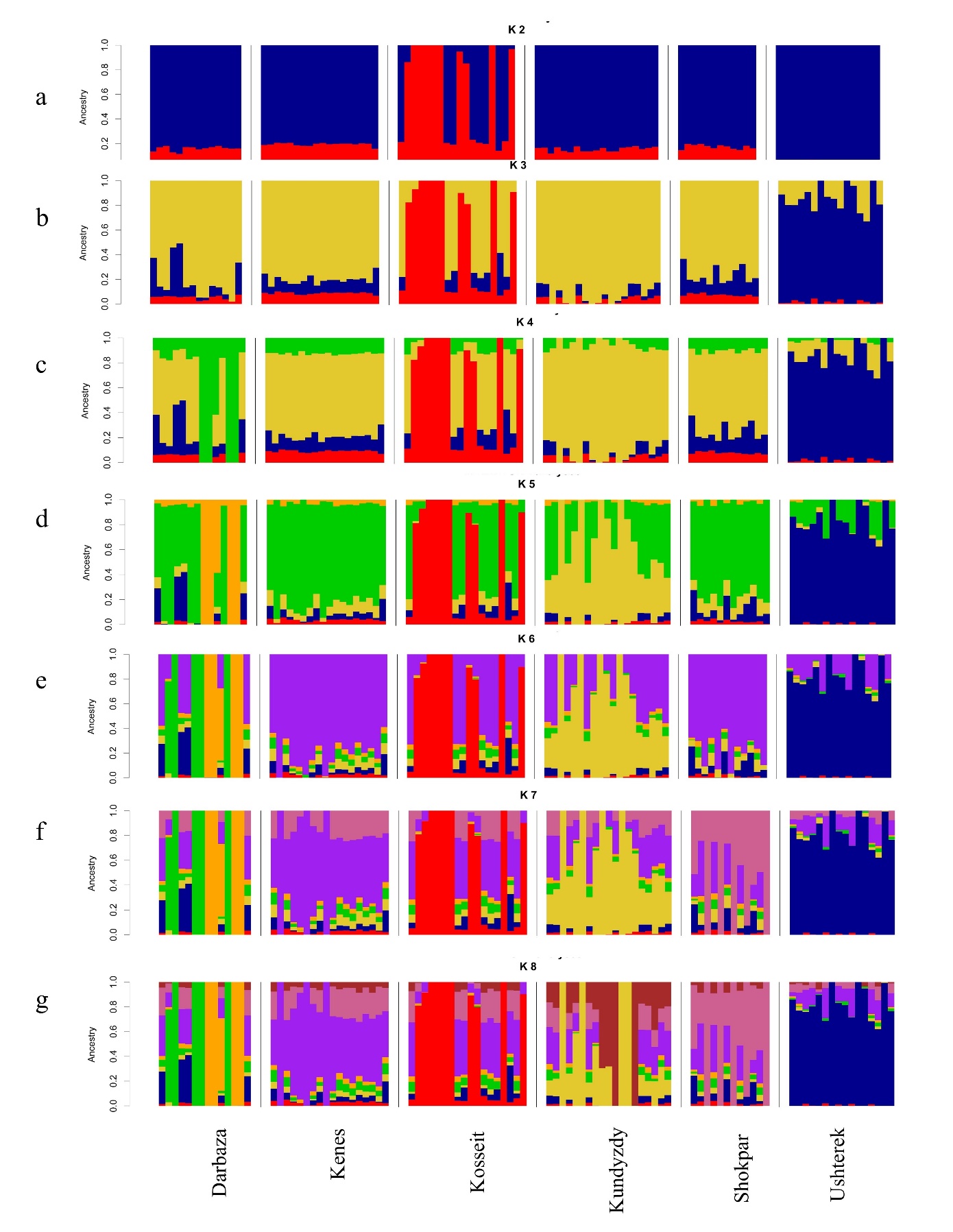
 **Figure S2:** Admixture analysis for Kazakhstan goats from six different regions showing the proportions of ancestral populations for K value from 2 to 8. Each vertical bar exemplifies an individual


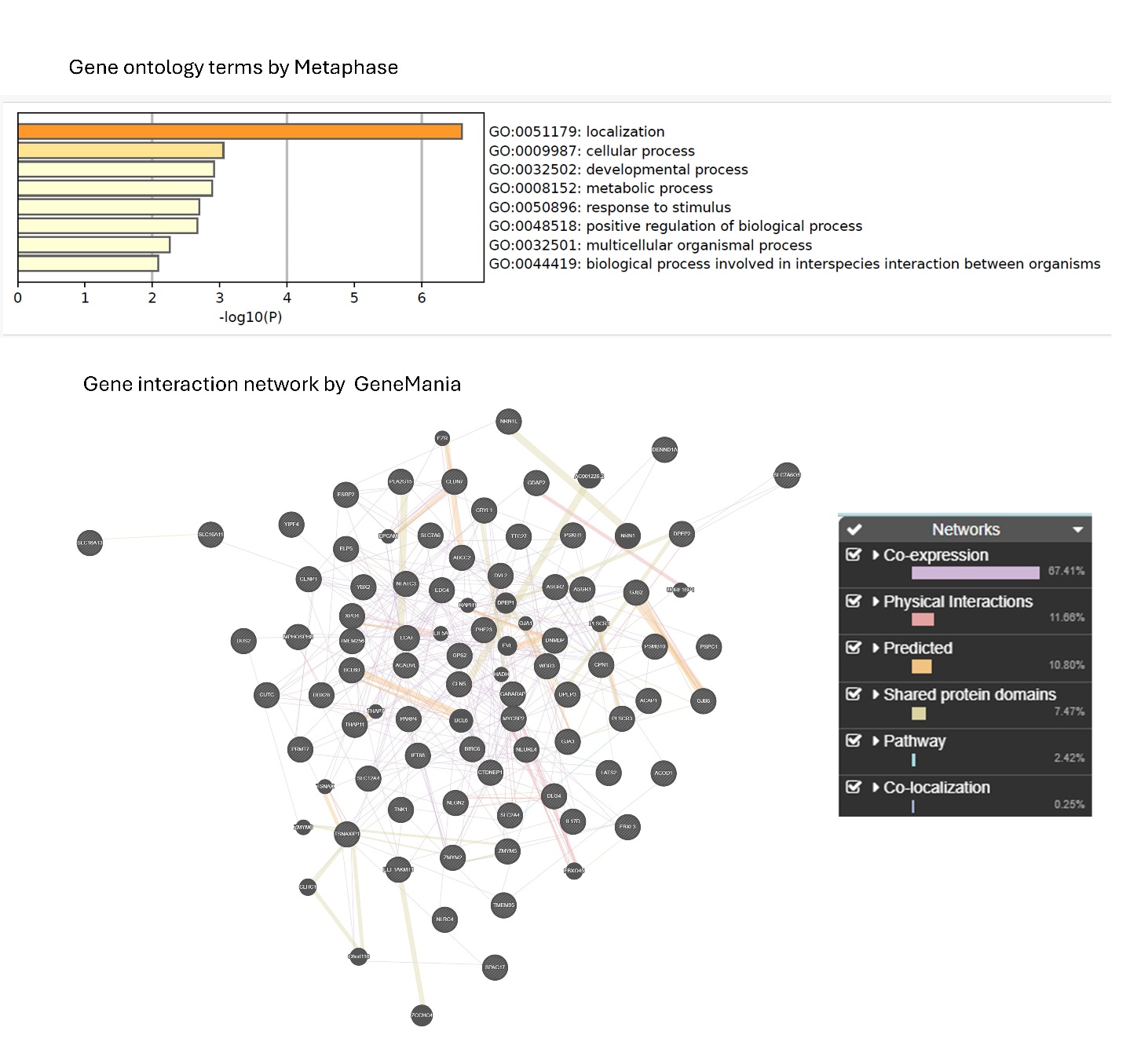


b

a

**Figure S3:** (a) Gene Ontology Enrichment (b) Gene Interaction Network for six goat population in Kazakhstan
